# The role of M1/M2 macrophage polarization in the pathogenesis of obesity-related kidney disease and related pathologies

**DOI:** 10.3389/fimmu.2024.1534823

**Published:** 2025-01-10

**Authors:** Periklis Dousdampanis, Ioanna Aggeletopoulou, Athanasia Mouzaki

**Affiliations:** ^1^ Department of Nephrology, Saint Andrews General Hospital of Patras, Patras, Greece; ^2^ Laboratory of Immunohematology, Department of Internal Medicine, Medical School, University of Patras, Patras, Greece; ^3^ Division of Gastroenterology, Department of Internal Medicine, Medical School, University of Patras, Patras, Greece

**Keywords:** chronic kidney disease, cardiovascular disease, macrophages, M1, M2, obesity

## Abstract

Obesity is a rapidly growing health problem worldwide, affecting both adults and children and increasing the risk of chronic diseases such as type 2 diabetes, hypertension and cardiovascular disease (CVD). In addition, obesity is closely linked to chronic kidney disease (CKD) by either exacerbating diabetic complications or directly causing kidney damage. Obesity-related CKD is characterized by proteinuria, lipid accumulation, fibrosis and glomerulosclerosis, which can gradually impair kidney function. Among the immune cells of the innate and adaptive immune response involved in the pathogenesis of obesity-related diseases, macrophages play a crucial role in the inflammation associated with CKD. In obese individuals, macrophages enter a pro-inflammatory state known as M1 polarization, which contributes to chronic inflammation. This polarization promotes tissue damage, inflammation and fibrosis, leading to progressive loss of kidney function. In addition, macrophage-induced oxidative stress is a key feature of CKD as it also promotes cell damage and inflammation. Macrophages also contribute to insulin resistance in type 2 diabetes by releasing inflammatory molecules that impair glucose metabolism, complicating the management of diabetes in obese patients. Hypertension and atherosclerosis, which are often associated with obesity, also contribute to the progression of CKD via immune and inflammatory pathways. Macrophages influence blood pressure regulation and contribute to vascular inflammation, particularly via the renin-angiotensin system. In atherosclerosis, macrophages accumulate in arterial plaques, leading to chronic inflammation and plaque instability, which may increase the risk of CVD in CKD patients. This review focuses on the involvement of macrophages in CKD and highlights their role as a critical link between CKD and other pathologies. Targeting macrophage polarization and the ensuing macrophage-induced inflammation could be an effective therapeutic strategy for CKD and related diseases and improve outcomes for patients with obesity-related kidney disease.

## Introduction

Obesity is a widespread health problem in both developing and industrialized countries. It affects both adults and children, and it is estimated that over 60% of the adult population worldwide will be overweight by 2030 (1). According to a meta-analysis from the United States, obesity is associated with 24-33% of all kidney diseases (2).

Obesity is known to alter various physiological functions of metabolism and cause metabolic diseases such as insulin resistance and type 2 diabetes, hypertension, atherosclerosis and CVD (3). Obesity is also associated with CKD, which leads to increased mortality (2, 3), as it promotes CKD development either through the effects of diabetes and its complications (diabetic kidney disease) or through a direct effect leading to kidney damage (4, 5).

Proteinuria due to hyperfiltration or/and podocytopathy, local lipid accumulation, fibrosis and glomerulosclerosis characterize obesity-related CKD (5). Against this background, direct renal damage caused by obesity could be considered a particular form of focal segmental glomerulosclerosis, which, in contrast to diabetic nephropathy, progresses slowly to CKD (5). Obesity also causes various changes in adipose tissue and alters metabolic and endocrine functions by polarizing macrophages (6–8).

Macrophages are divided into two types: the pro-inflammatory M1 and the anti-inflammatory M2 macrophages. M1 and M2 macrophages have different functions that follow an “as-needed” pattern in healthy people. In contrast, in many diseases characterized by a disturbed metabolism, such as obesity, macrophages polarize into M1 macrophages, which leads to inflammation (6–8).

Kidney inflammation in earlier stages could be considered a protective response to kidney injury to allow repair of kidney tissue (9). However, this initial inflammatory response may lead to the development and progression of CKD which results from the involvement of activated effector cytotoxic CD8+ T cells (Tc cells) and helper CD4+ T cells (Th cells), mast cells, natural killer (NK) cells as well as macrophages/monocytes and dendritic cells (9, 10). In the early stages of CKD, regardless of the underlying etiology, the inflammatory response is activated by the production of various growth factors and pro-fibrotic cytokines by immune cells, leading to concomitant renal fibrosis (10).

CKD is characterized by increased oxidative stress (11). Macrophages are the main source of oxidative stress, as these cells are involved in the formation of nitrotyrosine, which is thought to be responsible for oxidative damage and inflammatory diseases (12).

Type 2 diabetes is considered a disease with dual etiology, namely metabolic and inflammatory (13). In recent years, chronic inflammation has gained increasing attention in relation to the development of diabetes. Interestingly, inflammation is thought to protect metabolism (13). Hotamisligil et al. (14) were the first to suggest that inflammation is associated with metabolic disease. They reported that there is an overproduction of TNF-α in obese rats and that neutralization of TNF-α leads to increased glucose uptake in response to insulin. In this study, the authors reported that blockade of only one pro-inflammatory cytokine (TNF-α) can increase insulin sensitivity. In addition, Weisberg et al. (15) reported that obesity leads to increased infiltration of TNF-α-producing macrophages in adipose tissue.

Inflammation in hypertension is mediated by macrophages via activation of the renin-angiotensin system (RAS) (16). There is evidence that macrophages are involved in the inflammatory processes in the vascular system and interact with the arterial wall; furthermore, they are involved in the organ damage caused by high blood pressure and thus promote CKD (16).

It is known that atherosclerosis is also characterized by chronic inflammation resulting from an imbalance in lipid metabolism in combination with a maladaptive immune response. In the pathogenesis of atherosclerotic plaque in the arterial wall, an accumulation of macrophages with inflammatory properties has been identified (17).

Obesity, type 2 diabetes and insulin resistance, hypertension and atherosclerosis contribute to the development and progression of both CVD and CKD (18). Both diseases are characterized by increased oxidative stress and inflammation (19). In addition, CKD patients have an increased risk of CVD and vice versa, suggesting that the immune system, and macrophages in particular, are a link between CKD and CVD.

## Types of macrophages

Macrophages are cells of the innate immune system and belong to the large family of mononuclear phagocytes. They are present in every human organ and play a crucial role in maintaining tissue integrity by removing necrotic cells and debris. They are also involved in the remodeling of the extracellular matrix and the replacement of dead cells by secreting regenerative growth factors (20). Macrophages are professional antigen-presenting cells that present antigenic peptides to both Tc and Th cells via the HLA I and II molecules expressed on their surface (21).

**Figure 1:**
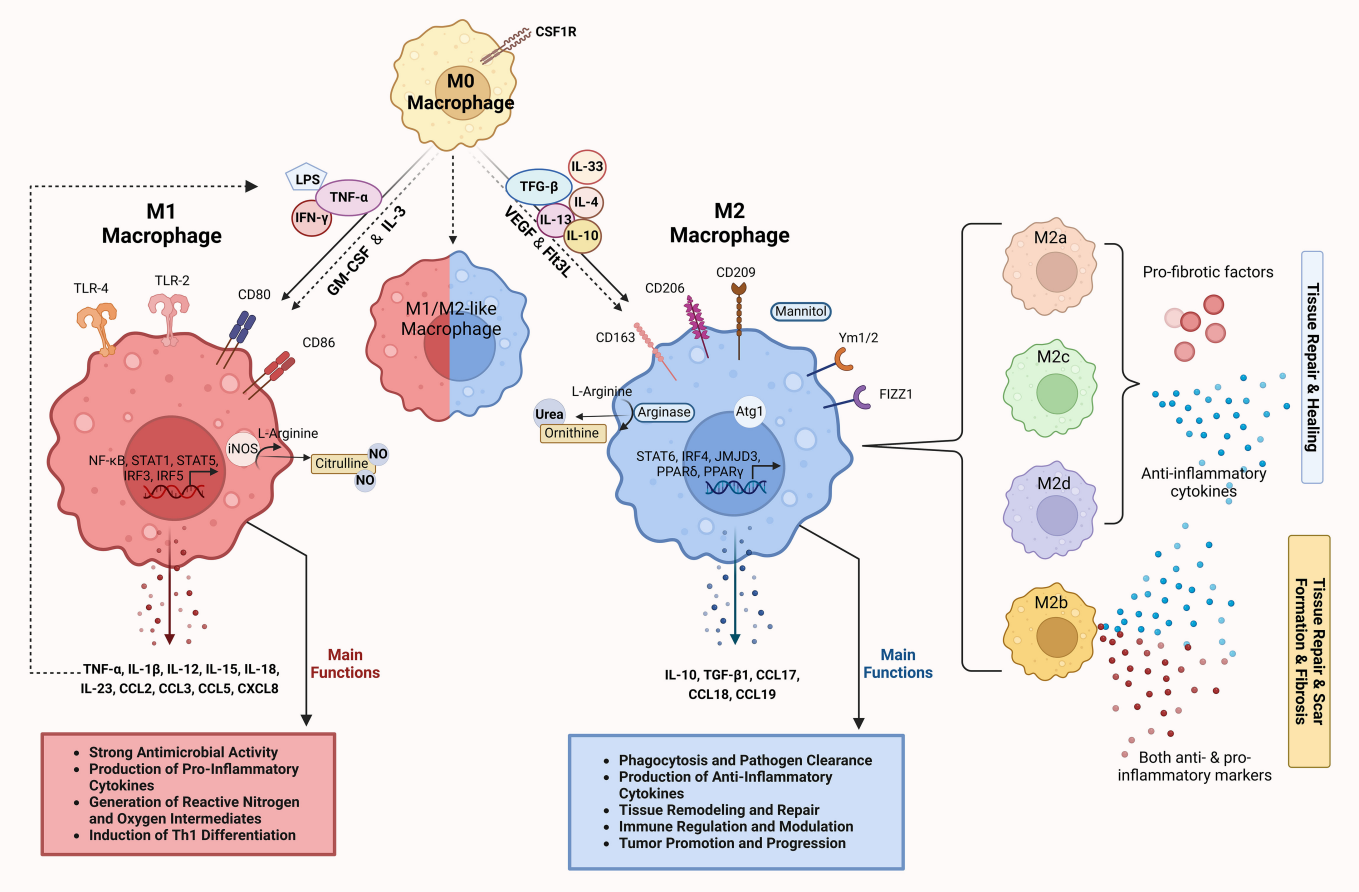
Differentiation and functions of macrophages along the M1/M2 polarization axis. M0 macrophages represent the undifferentiated or resting state of macrophages. Under the influence of CSF1R, M0 macrophages can differentiate into either M1 or M2 macrophages depending on the cytokine environment and the signaling molecules they encounter. M1 macrophages, induced by stimuli such as LPS and pro-inflammatory cytokines (IFN-γ and TNF-α), express surface markers such as CD80 and CD86 (24). Key signaling pathways involved include TLR-4, NF-κB, STAT1, STAT5 and IRFs, which control the expression of inflammatory genes (25). M1 macrophages produce high levels of pro-inflammatory cytokines (TNF-α, IL-1β, IL-12, IL-15, IL-18, IL-23) and chemokines (CCL2, CCL3, CCL5, CXCL8) (13, 26–31). Through iNOS activity, they generate reactive nitrogen and oxygen species that have a strong antimicrobial effect and promote the differentiation of Th1 cells (28, 32). The main functions of M1 macrophages include pathogen clearance, production of pro-inflammatory cytokines and the generation of reactive oxygen/nitrogen species to fight infections (13, 26, 27, 33, 34). M2 macrophages induced by anti-inflammatory cytokines (IL-4, IL-10, IL-13, IL-33, TGF-β) are involved in tissue repair and immunomodulation. They express surface markers such as CD163, CD206, CD209, Ym1/2 and Fizz1, which are involved in pathways such as STAT6, IRF4, JMJD3, PPARγ and PPARδ, and promote anti-inflammatory and tissue-remodeling functions (13, 26–28). Mannitol serves as a signaling molecule that can enhance M2 macrophage activation and function. M2 macrophages produce anti-inflammatory cytokines and chemokines (IL-10, TGF-β1, CCL17, CCL18, CCL19) and promote tissue repair through arginase activity, which converts L-arginine to urea and ornithine (26–31, 35, 36). The main functions of M2 macrophages include phagocytosis, pathogen clearance, tissue remodeling and immune regulation (13, 26, 27, 33, 34). The intermediate or hybrid state between M1 and M2 (M1/M2-like macrophage) represents macrophages with mixed functional properties. These macrophages exhibit features of both M1 and M2 polarization and play a complex role in immune regulation and tissue response. M2 macrophages are further divided into subtypes (M2a, M2b, M2c and M2d), each associated with distinct cytokine profiles and roles in tissue remodeling, as indicated by different colors (26, 35, 37). The dashed arrows show the reversible transitions between the polarization states and illustrate the plasticity of the macrophages. The image was created with Biorender (https://biorender.com).

The tissue microenvironment regulates the differentiation of precursor monocytes into macrophages. Macrophages differentiate into distinct functional populations, M1 and M2, in response to microbial products, stimulated lymphocytes or damaged cells in their microenvironment (7), noting that between the two ends of the M1 and M2 macrophage spectrum, there are macrophages with intermediate phenotypes characterized by their surface markers, secretome and functions (22, 23). The polarization and function of macrophages along the M1/M2 spectrum is depicted in **Figure 1**.

M1 macrophages are characterized by their strong antimicrobial properties and the production of pro-inflammatory cytokines, reactive nitrogen and oxygen intermediates as well as the induction of Th1 differentiation of Th0 cells. M2 macrophages are characterized by the most efficient phagocytosis and their involvement in tissue remodeling, immune regulation and tumor promotion (13, 26, 27, 33, 34).

Lipopolysaccharide (LPS) and the Th1 cytokines IFN-γ and TNF-α induce the generation of M1 macrophages. M1 macrophages express toll-like receptors (TLR) 2 and 4, CD80, CD86 and inducible nitric oxide synthase (iNOS). M1 polarization depends on the IRF/STAT, LPS/TLR and NF-κB/PI-3K pathways (24). The main transcription factors that regulate the gene expression profile associated with M1 polarization are NF-κB, AP-1, C/EBP-α, STAT1, STAT5, IRF3 and IRF5. TLR-mediated M1 polarization occurs through the activation of NF-κβ, AP-1, C/EBP-α, STAT1 and IRF5 (25). M1 macrophages mainly secrete TNF-α, but also other cytokines such as IL-1β, IL-12, IL-15, IL-18 and IL-23, as well as various chemokines such as CCL2, CCL3, CCL5 and CXCL8. These cytokines and chemokines induce further M1 polarization in a positive feedback loop (13, 26–31).

The cytokines IL-4, IL-10, IL-13, IL-33 and TGF-β induce the polarization of macrophages towards an M2 phenotype. M2 macrophages express the mannitol receptor, CD163, CD206, CD209, Atg1, Fizz1 and Ym1/2. The main transcription factors that regulate the gene expression profile associated with M2 polarization are STAT6, IRF4, JMJD3, PPARδ and PPARγ (13, 26–28). M2 macrophages mainly secrete IL-10 and TGF-β1 as well as the chemokines CCL17, CCL18 and CCL19 (26–31, 35, 36).

In addition, macrophage polarization is regulated by two opposing pathways in arginine metabolism: The iNOS pathway, which produces citrulline and NO from arginine, induces the generation of M1 macrophages, whereas the arginase pathway, which converts arginine to ornithine and urea, induces the generation of M2 macrophages (28, 32).

M2 macrophages are further subdivided into M2a, M2b, M2c and M2d subpopulations, all of which express anti-inflammatory and pro-fibrotic factors and are involved in tissue repair, with the exception of M2b macrophages, which can express both pro- and anti-inflammatory markers and are either involved in tissue repair or mediate scarring and tissue fibrosis (26, 35, 37).

The term “polarization” refers to the transition from the M1 to the M2 phenotype and vice versa (M1/M2 axis) (26). The process of M1/M2 polarization has been elucidated by studies in which macrophages were stimulated *in vitro* with different combinations of cytokines. According to these studies, the transmembrane tyrosine kinase receptor of mononuclear cells, colony stimulating factor 1 receptor, is an important regulator of macrophage polarization (38). Granulocyte macrophage colony stimulating factor (GM-CSF), IL-3, vascular endothelial growth factor (VEGF) and Fms-like tyrosine kinase 3 ligand (Flt3L) are also involved in macrophage differentiation and polarization (7, 39, 40).

## Macrophages and CKD

### Macrophages - obesity and diabetes

The link between obesity, insulin resistance and type 2 diabetes is well known (41). It should be emphasized that central obesity is one of the most important predisposing factors for type 2 diabetes. Central obesity or abdominal obesity reflects increased visceral fat. According to the WHO criteria, central obesity is defined as a waist circumference ≥94 cm in men and ≥80 cm in women. Furthermore, central obesity is involved in adipocyte-macrophage polarization (42). In obesity, increased production of proinflammatory cytokines, free fatty acids, monocyte chemoattractant protein 1 (MCP-1) and CCL2 leads to macrophage polarization in adipose tissue to the proinflammatory M1 phenotype (43, 44). These adipose tissue macrophages mainly secrete TNF-α, IL-6 and IL-1β (8); they also trigger NF-κB activation via P62 and peroxisome proliferator-activated receptor γ (PPARγ) (45). PPARγ promotes further M1 macrophage polarization and the production of pro-inflammatory cytokines, which is catastrophic for islet cells (45). The presence of macrophages in the kidneys is associated with exacerbation of diabetic nephropathy (46).

Obese people are characterized by severe adipose tissue inflammation, insulin resistance and glucose intolerance. The increased release of adipocyte chemokines, including MCP1 and leukotriene B4 (LTB4), is responsible for the transport and increased accumulation of immune and adaptive immune cells in adipose tissue (47, 48). LTB4 in particular, leads to inflammation and insulin resistance in obesity (48). M1 adipose tissue macrophages express several surface markers, including CD11b, CD11c and F4/80 (15, 44). This process requires energy derived from anaerobic glycolysis rather than oxidative phosphorylation. Moreover, anaerobic glycolysis enhances the polarization of macrophages in adipose tissue towards the M1 phenotype (49). Interestingly, a decrease in oxidative phosphorylation in the mitochondria leads to a further increase in the production of reactive oxygen species (ROS) (50).

Both insulin resistance and β-cell dysfunction are implicated in the underlying disease process of diabetes (30). Macrophages play a critical role in the normal development of β-cells during embryogenesis and can promote β-cell replication in experimental models of pancreatic regeneration (31, 51).

Data from animal studies show that tissue migration of macrophages is responsible for the pro-inflammatory state that precedes and leads to insulin resistance (31). Islet inflammation and β-cell dysfunction in type 2 diabetes are mainly caused by the production of IL-1β by islet macrophages (30) and the islets themselves (52). Increased numbers of immune cells in the islets of patients with type 2 diabetes also contribute to the low-grade islet inflammation associated with the development of type2 diabetes (53). In addition, adipose tissue inflammation contributes to insulin resistance through the production of TNF-α and IL-6 (53). Mitogen-activated protein kinases trigger apoptosis of β-cells by inducing apoptotic signaling from the mitochondria of diabetic patients (54).

The pathogenic mechanism of islet cell inflammation is complex. Infiltration of immune cells, release of cytokines, apoptosis of β-cells, deposition of amyloid and fibrosis of islet cells are involved in the pathogenesis of islet cell inflammation. In particular, high glucose or palmitate induce islet cell cytokine secretion and promote the migration of monocytes and neutrophils. In addition, macrophages and NK cells have an increased capacity for phagocytosis, chemotaxis and cytokine/chemokine secretion (4, 51, 55).

Macrophage-dependent insulin resistance leads to a decrease in glycogen synthesis and muscle protein catabolism (30). In addition, insulin resistance leads to increased lipoprotein lipase activity in adipocytes with a concomitant increase in free fatty acids.

The increased secretion of cytokines from islet cells leads to a decrease in insulin sensitivity and a change in glucose metabolism (30). Islet cell failure is characterized by inefficient activation of glucose-stimulated insulin production and impaired suppression of glucagon release (30).

Data from experimental studies suggest that the initial protective inflammatory response catalyzing insulin resistance depends on tissue macrophages (30). Furthermore, according to studies using experimental models of diabetic nephropathy, there is clinical evidence that diabetic nephropathy is associated with an increase in macrophage infiltration due to upregulation of MCP-1 and intracellular adhesion molecule 1 (ICAM-1) (30). In the context of diabetic nephropathy, macrophage accumulation is associated with exacerbation of CKD, suggesting that inflammation is the underlying mechanism of the disease (56). Interestingly, genetic deficiency of ICAM-1 and/or MCP-1 protects against diabetic nephropathy by reducing the number of renal macrophages (57).

High glucose, advanced glycation end products and oxidized low-density lipoprotein induce the production of cytokines by tissue macrophages, including IL-1, TNF-α, and fibrosis transforming growth factor and thrombosis tissue factor (58). In addition, macrophages contribute to renal fibrosis by producing ROS and metalloproteinases (58). The deposition of AGEs in several involved tissues in the milieu of type 2 diabetes may contribute to the macrophage-mediated tissue damage in diabetic complications including renal injury (56). AGEs are also involved in the inflammatory process by stimulating macrophage activation and secretion of various cytokines through interaction with their receptor (RAGE).

Macrophage-derived TNF-α and NO are both cytotoxic and involved in vascular smooth muscle cell apoptosis and atherosclerotic plaque instability (56). In addition, IL-1β and TNF-α derived from islet macrophages induce Fas receptor (FasR) expression, leading to apoptosis of β-cells through FasR death signaling (59, 60).

In addition, macrophages from patients with type 2 diabetes have an increased number of NLRP3s, leading to activation of the NLRP3 inflammasome (61). NF-κB triggers the activation of the NLRP3inflammasome, which in turn increases the release of IL-1β, IL-18 and caspase I, and leads to β-cell death (62, 63).

All in all, the increased infiltration of adipose tissue macrophages observed in animal models and in humans with diabetes suggests that adipose tissue is one of the main sites of the chronic inflammatory response associated with obesity and type 2diabetes.

### Macrophage polarization and renal fibrosis

Fibrosis is the pathophysiological alteration of the kidneys in response to endogenous or/and exogenous stimuli, leading to a marked reduction in glomerular filtration rate and deterioration of renal function (64).

It is well known that inflammation plays an important role in renal fibrosis (65). Activation of fibroblasts by the release of various inflammatory mediators and infiltration of immune cells at the site of renal injury leads to collagen synthesis (64, 65). The accumulation of collagen in the injured tissue promotes sclerosis of the renal parenchyma with the formation of scars and eventually complete loss of renal function (64).

Renal fibrosis is characterized by three phases: the first phase of inflammation, the second phase of fibrosis mediated by fibroblasts and collagen production, and the final phase of scar formation.

In the context of renal injury, GM-CSF, LPS, IL-1, TNF-α, ιFN-γ, and various endogenous damage-associated molecular patterns (DAMPs) released by the damaged renal cells promote the polarization of macrophages to the M1 phenotype (65–67). M1 macrophages induce the activation of metalloproteinases and oxidative stress through the production of NO and ROS (68). In addition, the increased production of endothelin due to M1-induced secretion of IL-1 and TNF-α alters hemodynamics (69). Hypoxia of the renal microenvironment induces macrophages to produce pro-inflammatory and pro-angiogenic factors (70).

The number of M2 macrophages is decreased in the early and middle stages of renal injury, whereas it is increased in the end-stage (71). M2 macrophages have an anti-inflammatory and injury-healing function (72). These effects are mediated by the secretion of IL-10, TGF-β, VEGF and EGF (71, 73). More specifically, M2 suppress the inflammatory response and promote tissue remodeling (74, 75). Activation of the IL-10R on M2 macrophages decreases the secretion of inflammatory factors while increasing the production of anti-inflammatory factors, leading to regression of tissue inflammation. Data from experimental studies show that IL-10R signaling can suppress the secretion of the pro-inflammatory mediator IL-23. When IL-10R signaling is impaired, there is increased secretion of IL-23, leading to neutrophil activation and an enhanced inflammatory response (76).

Although M2 macrophages contribute to tissue repair, they can also promote fibrosis. The presence of CD206+/CD163+ M2 macrophages at the site of renal injury is associated with the severity of fibrosis (66). TGF-β stimulates M2a macrophages by activating the ATF6/TGF-β/SMAD3 pathway, leading to the activation of macrophage migration inhibitory factor (MIF) that induces the transition of macrophages to myofibroblasts in renal fibrosis (66, 77). In this regard, regulating the effect of TGF-β on macrophages could be a challenge in the treatment of CKD at earlier stages. In addition, IL-4 and IL-13 activate the JAK1 and JAK3 signaling pathways with concomitant activation of STAT3, leading to renal fibrosis (78).

Injured renal epithelial cells produce DAMPs and pathogen-associated molecular patterns (PAMPs) (79). Tissue-resident macrophages recognize these patterns via pattern recognition receptors (PRRs), become activated and produce cytokines, which in turn recruit macrophages from the bone marrow (79); this process leads to M1 polarization of the macrophages involved, regardless of their origin (80, 81).

Interestingly, increased migration of macrophages around tubular epithelial cells that have been damaged by lipotoxicity has been described as a hallmark of diabetic nephropathy (66, 82). However, the interaction and relationship between these cells has not yet been clarified. In an experimental model of diabetic nephropathy, tubular epithelial cells can release extracellular vesicles containing leucine-rich alpha-2-glycoprotein 1 (LGR1) and activate macrophages. Conversely, stimulated macrophages can also secrete extracellular vesicles that promote apoptosis of tubular epithelial cells (83). In addition, stimulation of renal tubular epithelial cells by external factors leads to upregulation of TNF-α, ιL-1β and IL-6, which is associated with the polarization of macrophage to M1 after renal injury (84).

Increased expression of CD74 has been found in podocytes and parietal epithelial cells (85). CD74 is a receptor for MIF. The interaction of MIF with CD74 stimulates the production of inflammatory cytokines by podocytes and the proliferation of parietal epithelial cells (85). Interestingly, in the context of polycystic kidney disease, MIF is considered a potent regulator of cyst growth by inhibiting the AMP-activated protein kinase signaling pathway (86).

**Figure 2:**
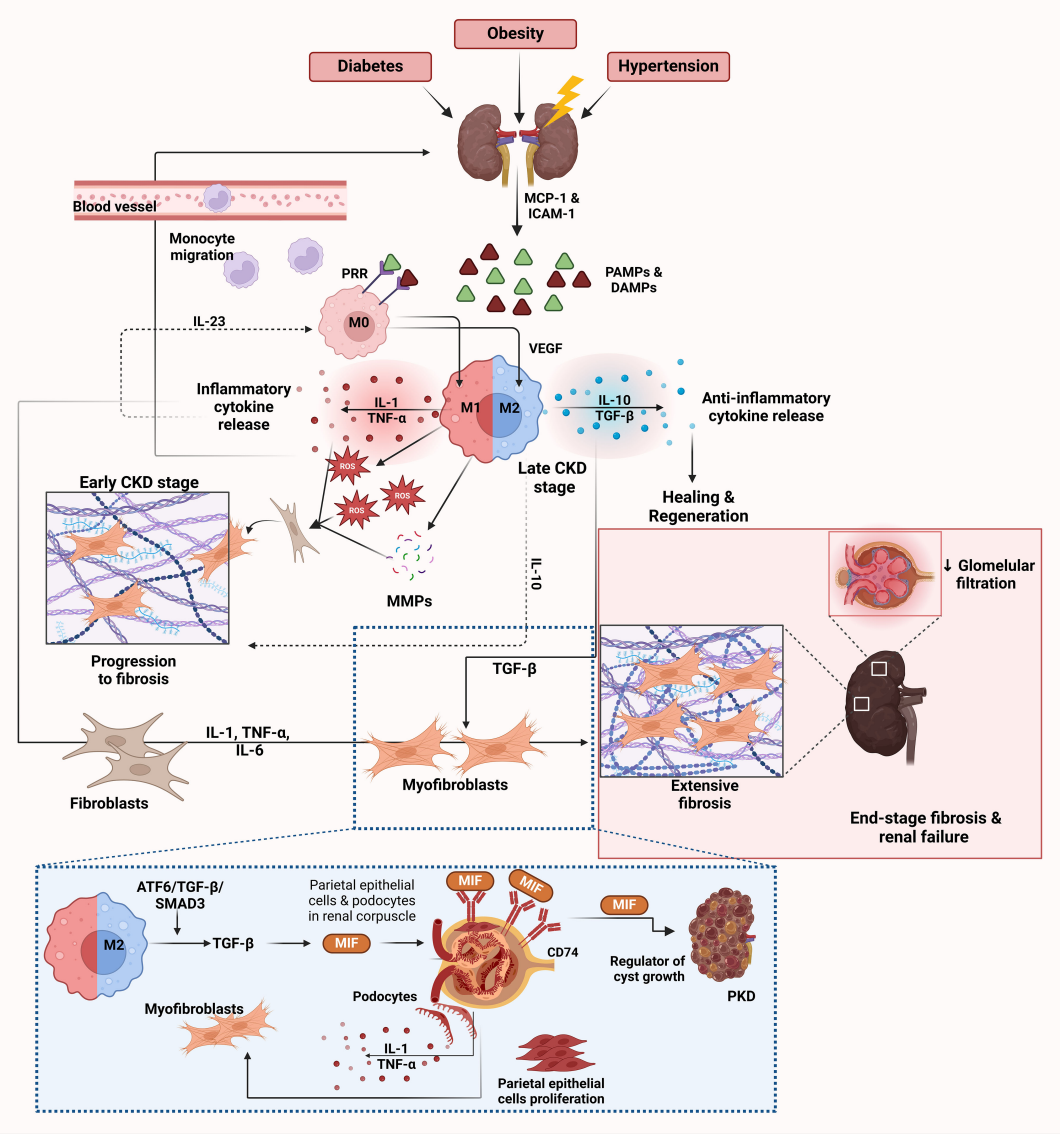
Role of macrophages in CKD. CKD is triggered by various conditions such as diabetes, obesity and hypertension, leading to renal injury. This initial damage results in the release of DAMPs and PAMPs from injured kidney cells (65–67, 79). These molecules activate endothelial cells to release chemokines such as MCP-1 and ICAM-1, which facilitate the recruitment and migration of monocytes from the bloodstream into the kidney, where they differentiate into M0 macrophages (30). PRRs on M0 macrophages recognize DAMPs and PAMPs and initiate further macrophage activation and polarization (79–81). After activation by PRRs, macrophages polarize towards the M1 phenotype in the early stages of CKD. M1 macrophages release inflammatory cytokines such as IL-1 and TNF-α together with ROS and MMPs (68–70). These molecules exacerbate kidney injury by promoting inflammation and further damaging renal tissue. This release of inflammatory cytokines creates a feedback loop that recruits more immune cells and perpetuates the inflammatory environment in the kidney, as depicted by the arrows in the inflammatory cytokine release cycle. Chronic inflammation due to M1 macrophage activity and the persistent presence of ROS and MMPs leads to fibroblast activation (64, 65). Activated fibroblasts differentiate into myofibroblasts and produce excessive amounts of extracellular matrix proteins such as collagen, leading to tissue scarring (64, 65). As CKD progresses, there is a shift toward the M2 macrophage phenotype to resolve inflammation and promote tissue healing (72). M2 macrophages release anti-inflammatory cytokines, primarily IL-10 and TGF-β, which suppress inflammation and facilitate healing (71, 73). However, despite their role in healing, M2 macrophages also contribute to fibrosis by promoting collagen deposition through the release of VEGF and EGF, which enhances tissue regeneration but can lead to excessive fibrosis (71, 73). The dashed lines highlight the cycle of inflammation and fibrosis driven by IL-10 and IL-23, which perpetuates macrophage polarization and cytokine release (76). IL-23 enhances the M1 macrophage response, while IL-10 in the M2 response can lead to prolonged inflammation and fibrosis, creating a chronic inflammatory environment (76). The ATF6/TGF-β/SMAD3 pathway in M2 macrophages leads to activation of MIF, which plays a central role in renal fibrosis (66, 77). MIF acts via CD74, a receptor expressed on podocytes and parietal epithelial cells in the renal corpuscle (85). The interaction between MIF and CD74 stimulates the production of inflammatory cytokines in podocytes, including IL-1 and TNF-α, and triggers proliferation of parietal epithelial cells (85). This contributes to inflammation, fibrosis, and kidney damage. In the context of polycystic kidney disease (PKD), MIF also acts as a regulator of cyst growth, which further exacerbates disease progression (86). End-stage CKD is characterized by extensive fibrosis and scarring of renal tissue, leading to decreased glomerular filtration rate and loss of kidney function (64). The inflammatory cytokines IL-1, TNF-α and IL-6 contribute to the activation of fibroblasts and differentiation into myofibroblasts and promote the progression of fibrosis (66, 77). Endothelin and TGF-β also play a role in fibroblast activation and collagen deposition, which further drives fibrosis (69). Markers of fibrosis are associated with activated macrophages and myofibroblasts in the fibrotic kidney. This eventually leads to end-stage renal disease, depicted in the figure by the extensive scarring and fibrosis in the kidney tissue. The image was created with Biorender (https://biorender.com).

**Figure 2** illustrates the sequential role of macrophages in the progression of CKD, emphasizing key stages, cytokine interactions, and molecular pathways.

## Macrophage involvement in CVD

### Macrophages and hypertension

Obesity is a risk factor for hypertension and CVD (87). Uncontrolled hypertension is known to cause end organ damage, including heart failure, myocardial infarction, CKD and stroke (88).

The immune system is involved in the pathogenesis of hypertension (89–91). As early as the 1950s, Robert Hodgson Heptinstall reported an infiltration of leukocytes in renal biopsies from hypertensive patients (92). In addition, the severity of nephrosclerosis was associated with the extent of leukocyte accumulation (93). Data from animal studies suggest that in addition to leukocytes, other immune cells such as myeloid cells are also found in the kidneys in hypertension (94). Interestingly, transfer of immune cells from animals with hypertension to animals with normal blood pressure caused hypertension in the normotensive animals (95).

Modulation of the immune response with immunosuppressants may interfere with the RAS system and prevent organ damage (96, 97). There is evidence that macrophages are involved in the pathogenesis of RAS-induced hypertension and subsequent organ damage, including renal damage (98, 99).

Circulating monocytes from hypertensive patients exhibit an increased pro-inflammatory phenotype compared to normotensive controls, and sera from these hypertensive patients have increased levels of pro-inflammatory cytokines (100). These macrophage-produced cytokines and ROS are involved in the pathogenesis of hypertension-induced endothelial dysfunction and the resulting decreased renal sodium excretion (89).

Activated macrophages and monocytes express lysozyme M (LysM+). Studies using conditional knockout LysM+-Cre mice have shown that depletion of monocytes expressing LysM+ improves blood pressure, vascular dysfunction, and smooth muscle dysfunction and prevents vascular ROS formation in the chronic angiotensin II (Ang II) infusion model (101). In addition, LysM+ monocytes increase vascular oxidative stress and blood pressure levels (101). Although not all macrophages are derived from circulating monocytes, under inflammatory conditions such as hypertension, all macrophages are derived from circulating monocytes (102).

Interestingly, macrophages in the skin can inhibit the sensitivity of salt-sensitive hypertension by stimulating lymphangiogenesis (103). Data from experimental studies report that in salt-sensitive hypertension, high sodium intake leads to increased hypertonic sodium accumulation in the skin and increased macrophage migration at this site (104–106). Skin macrophages release VEGF-C via a TonEBP(NFAT5)-dependent mechanism (107). In addition, VEGF-C binds to VEGFR3 and induces hyperplasia of the cutaneous lymphatic capillary network (107). In particular, blockade of this immune-induced lymphatic response leads to increased electrolyte accumulation in the skin and the development of salt-sensitive hypertension. In addition, TonEBP mediates NOS2-dependent generation of NO, and NO induces local vasodilation and renal sodium excretion (108). Furthermore, activated macrophages release TonEBP. The involvement of TonEBP in the renal tubules in the regulation of salt-sensitive blood pressure and urinary sodium transport is well established. Interestingly, TonEBP in renal macrophages limits renal tubular sodium re-absorption (109).

Cyclooxygenase-2 (COX2) in macrophages is also involved in the regulation of salt-sensitive hypertension by inducing not only the production of natriuretic prostaglandins but also VEGF-C-dependent lymphangiogenesis (110).

Interestingly, increased sodium intake can influence macrophage polarization (111). There is evidence that sodium retention increases blood pressure not only by volume overload, but also by influencing macrophage polarization to promote renal and vascular injury (112).

Macrophages can also mediate end-organ damage through mechanisms independent of blood pressure. In this context, CCR2 deficiency in the Ang II hypertension model limits the accumulation of macrophages in the kidney and reduces local oxidative stress and renal fibrosis (113).

RAS activation induces the activation and migration of monocytes in the kidneys (114). Ozawa et al. (115) reported that chronic Ang II infusion causes migration and accumulation of macrophages in the renal interstitium, triggering the mechanism of renal fibrosis. Angiotensin type 1 receptor (AT1R) activation induces the migration of monocytes from the spleen into the subendothelial vessels, leading to vascular damage and atherosclerotic lesions (116, 117). Ang II is involved in the process of differentiation, migration and activation of proinflammatory monocytes in the early stages of myeloid cell development (118).

It appears that RAS activation triggers inflammation and AT1R activation has an anti-inflammatory and protective effect directly on myeloid cells (119, 120). AT1R activation on macrophages mitigates renal fibrosis during RAS activation by limiting macrophage polarization, rather than through a direct effect on blood pressure regulation (99).

Crowley et al. (121), using a renal cross-transplantation model, reported that AT1R activation mediates RAS-induced hypertension, cardiac hypertrophy and renal injury. Thus, activation of AT1Rs in the kidney leads to renal injury and triggers a secondary immune response through the production of TNF-α and IL-1β from local mononuclear cells of the damaged tissue (122). Intravenous infusion of these cytokines causes a reduction in blood pressure through natriuresis (123–125).

Global RAS activation induces the secretion of TNF-α by macrophages, T cells, renal cells of the epithelium and mesangium, and cardiac cells (126–128). TNF-α acts on its receptors TNF21 and TNFR1/2 and triggers the activation of various signaling pathways of renal fibrosis, including TGF-β, mitogen-activated protein kinase (MAPK), NF-κB and NADPH oxidase (129–132). In addition, TNF-α causes hypertension by decreasing renal sodium excretion and may cause renal damage through a direct effect inducing renal fibrosis or/and through the intermediate effect of hypertension (133).

IL-1 is also involved in the pathogenesis of hypertension and hypertension-induced organ damage (134). Hypertension can induce increased expression of IL-1β through various components of the NLRP3 inflammasome (135). In particular, deficiency of these components reduces Ang II-induced hypertension (136).

### Macrophages and atherosclerosis

Obesity is one of the most important predisposing factors for the development of atherosclerotic cardiovascular disease (87). Both subcutaneous and visceral obesity are metabolically active tissues involved in the pathogenesis of atherosclerosis (137). In particular, obesity leads to an increase in saturated fatty acids, resulting in oxidative stress and subsequent endothelial dysfunction and atherosclerosis (138).

Obesity is characterized by a decrease in anti-inflammatory/atheroprotective adipokines and an increase in proinflammatory/atherogenic adipokines (139). Several adipokines/cytokines such as leptin, resistin, retinol-binding protein 4 (RBP4), angiopoietin-like protein 2, IL-6 and MCP-1 promote the atherosclerotic process by inducing inflammation, endothelial dysfunction and insulin resistance (139). In contrast, adiponectin has an anti-inflammatory and atheroprotective effect (139, 140).

Atherosclerosis is a chronic inflammatory response. Macrophages are involved in the development of atherosclerotic plaques from plaque progression through calcification to rupture and regression (141). The early phase of plaque progression is characterized by leukocyte infiltration, lipid accumulation, expansion of the necrotic core and the formation of a fibrous cap (141).

The local production of CCL2, CCL5, CX3CL1 and CXCL12 promotes the migration of monocytes from the bloodstream into the vessels and especially transepithelial migration in the vessel wall (142). Interestingly, the stage of plaque macrophage progression depends on the intensity of monocyte myelopoiesis and macrophage proliferation, the increased expression of chemokines and the expression of several inhibitor molecules for migration within the plaque, including netrin-1 (142).

IL-8, IL-12 and IL-18 produced by plaque macrophages induce plaque progression (143–145). M1 macrophages are predominant in progressive plaques and they induce chronic inflammation of plaques by releasing IL-1, IL-6 and TNF-α (141, 146, 147). Activation of the pro-inflammatory NLRP3 inflammasome/IL-1 axis induces plaque progression and thrombosis through monocyte recruitment, endothelial cell activation and angiogenesis (148, 149). IL-6 promotes proliferation of vascular smooth muscle cells, thrombosis and lipid accumulation in macrophages (150). TNF-α induces endothelial dysfunction, decreases NO bioavailability and increases ROS production (151).

M2 macrophages exert an anti-inflammatory effect and inhibit the formation of a necrotic core by secreting the anti-inflammatory cytokines IL-10 and TGF-β (152, 153). IL-10 exerts its anti-inflammatory effect by inhibiting the expression of several pro-inflammatory cytokines, metalloproteinases such as MMP-9, COX2 and several apoptosis inhibitors such as caspase-3 (150, 154). TGF-β stabilizes plaques through several mechanisms, including reduction of inflammation, cholesterol efflux from macrophages, and induction of collagen production (155, 156). Nevertheless, not all M2 macrophages exert anti-inflammatory effects. CD163+ M2 macrophages have been shown to promote angiogenesis and enhance leukocyte migration and permeability, thereby contributing to plaque progression (157, 158).

Macrophages form foam cells through the uptake of apolipoprotein B-containing lipoproteins (159). These foam cells induce endoplasmic reticulum stress, apoptosis and the secretion of various MMPs involved in the expansion of the necrotic core (160, 161).

Scavenger receptors (SRs) (162) expressed on macrophages are also associated with atherosclerosis. SRs bind and endocytose acetylated or oxidized (modified) LDL but not native LDL. Modified LDL accumulates in plasma and blood vessel walls and is considered to be a DAMP. Although functional loss of SRs can result in hypercholesterolemia, which leads to atherosclerosis and heart disease, it has been shown that when the SR-B2 is exposed to modified LDL, it interacts with TLR4 and TLR6, resulting in NF-κB activation and contributing to the inflammatory response associated with atherosclerosis and promoting necrotic lesions of plaques (163, 164).

Macrophages can eliminate apoptotic cells by efferocytosis (165, 166). In this way, macrophages can limit the inflammatory response and prevent the progression of atherosclerosis.

M1 macrophages form microcalcifications in the plaques, which increase the risk of plaque rupture (167, 168). In contrast, M2 macrophages stabilize the atherosclerotic plaque by forming macrocalcifications mediated by IL-10 (169).

M1 macrophages promote osteogenic transdifferentiation of vascular smooth muscle cells by secretion of IL-1β and TNF-α with concomitant increased mineralization of plaques (170, 171).

Macrophages are also involved in plaque rupture by secreting various MMPs (172). MMPs thin the fibrous cap of the plaque by degrading collagen and elastin (173).

**Figure 3:**
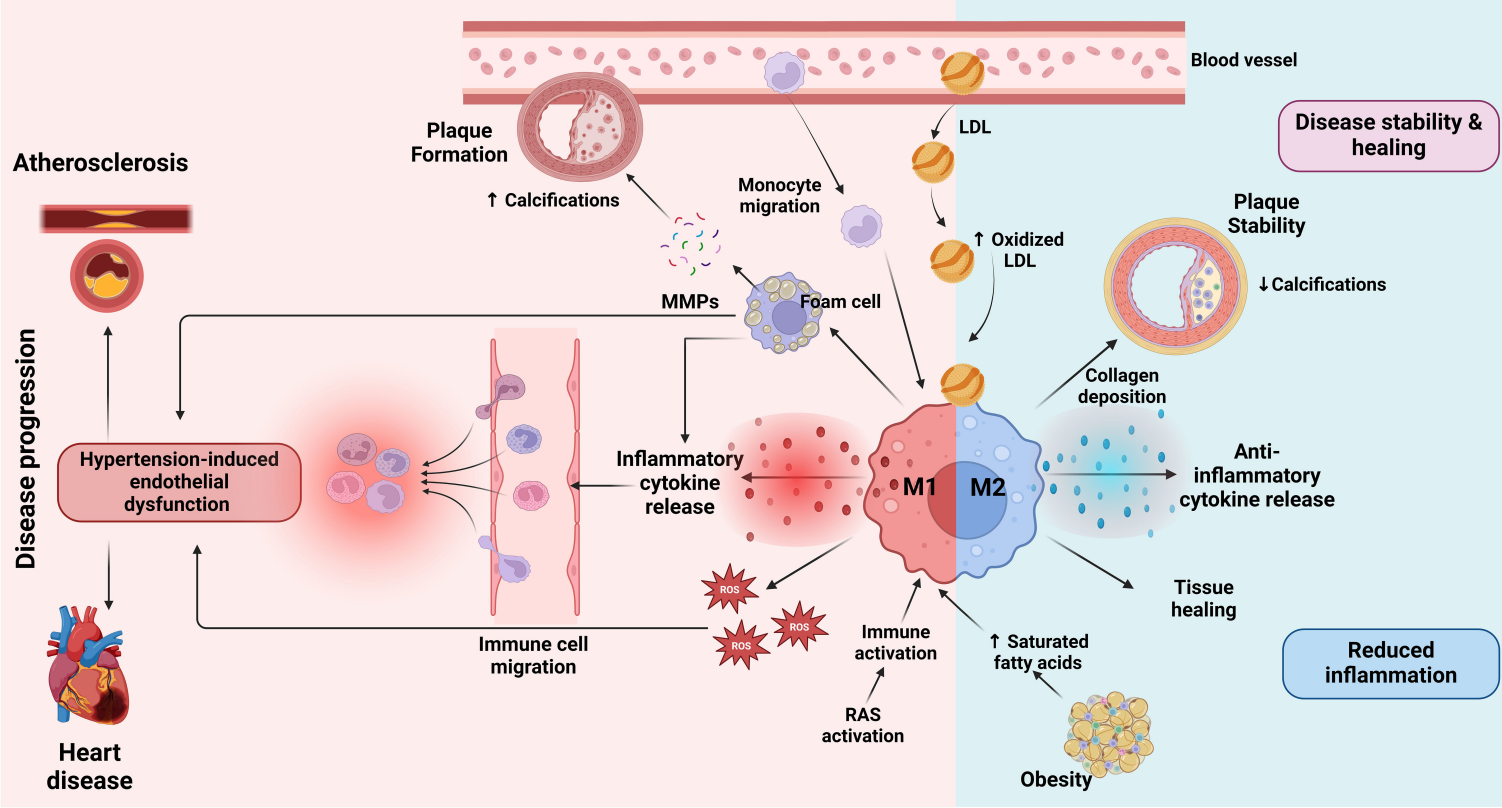
Role of macrophages in CVD. At the onset of atherosclerosis, LDL particles accumulate in the blood vessel walls, where they undergo oxidation to form oxidized LDL (163, 164). This triggers monocyte migration into the vessel wall, where they differentiate into macrophages. Upon activation by inflammatory stimuli, macrophages transition into the M1 state, releasing inflammatory cytokines and ROS which drive further vascular inflammation (89). Additionally, RAS activation contributes to immune activation and promotes an inflammatory response that exacerbates the progression of atherosclerosis (96–99). This inflammation is further amplified by hypertension-induced endothelial dysfunction, which facilitates immune cell migration into the vessel wall and promotes plaque instability (56). Factors such as obesity and saturated fatty acid accumulation may influence macrophage polarization towards M1, contributing to a more unstable plaque environment (138). M1 macrophages contribute to plaque formation by ingesting oxidized LDL and transforming into foam cells that promote the release of matrix MMPs (159). MMPs degrade extracellular matrix components, weakening the plaque and promoting calcifications within it, which can lead to plaque rupture (160, 161). The presence of M1 macrophages is therefore associated with increased inflammation, plaque vulnerability and progression of CVD, which may ultimately lead to heart disease (167, 168). Conversely, M2 macrophages, which are activated in response to anti-inflammatory signals, play a role in disease stability and healing (169). M2 macrophages secrete anti-inflammatory cytokines that attenuate inflammation, promote collagen deposition and stabilize the plaque by reducing calcifications (169). These macrophages support tissue healing and contribute to reduced inflammation in the vessel wall. The balance between M1 and M2 macrophage activation determines the overall inflammatory state of the atherosclerotic plaque and its stability, thus influencing the progression or attenuation of cardiovascular disease. The image was created with Biorender (https://biorender.com).

Inhibition of both the local production of macrophages and their migration from the bloodstream to the site of inflammation promotes the regression of atherosclerotic plaques (174, 175). In addition, increased HDL levels contribute to plaque regression by increasing cholesterol efflux and inducing macrophage polarization to anti-inflammatory M2 (176, 177). **Figure 3** illustrates the dual roles of M1 and M2 macrophages in CVD, focusing on their effects on plaque formation, vascular inflammation, and tissue remodeling.

## Conclusions and future prospects

**Figure 4:**
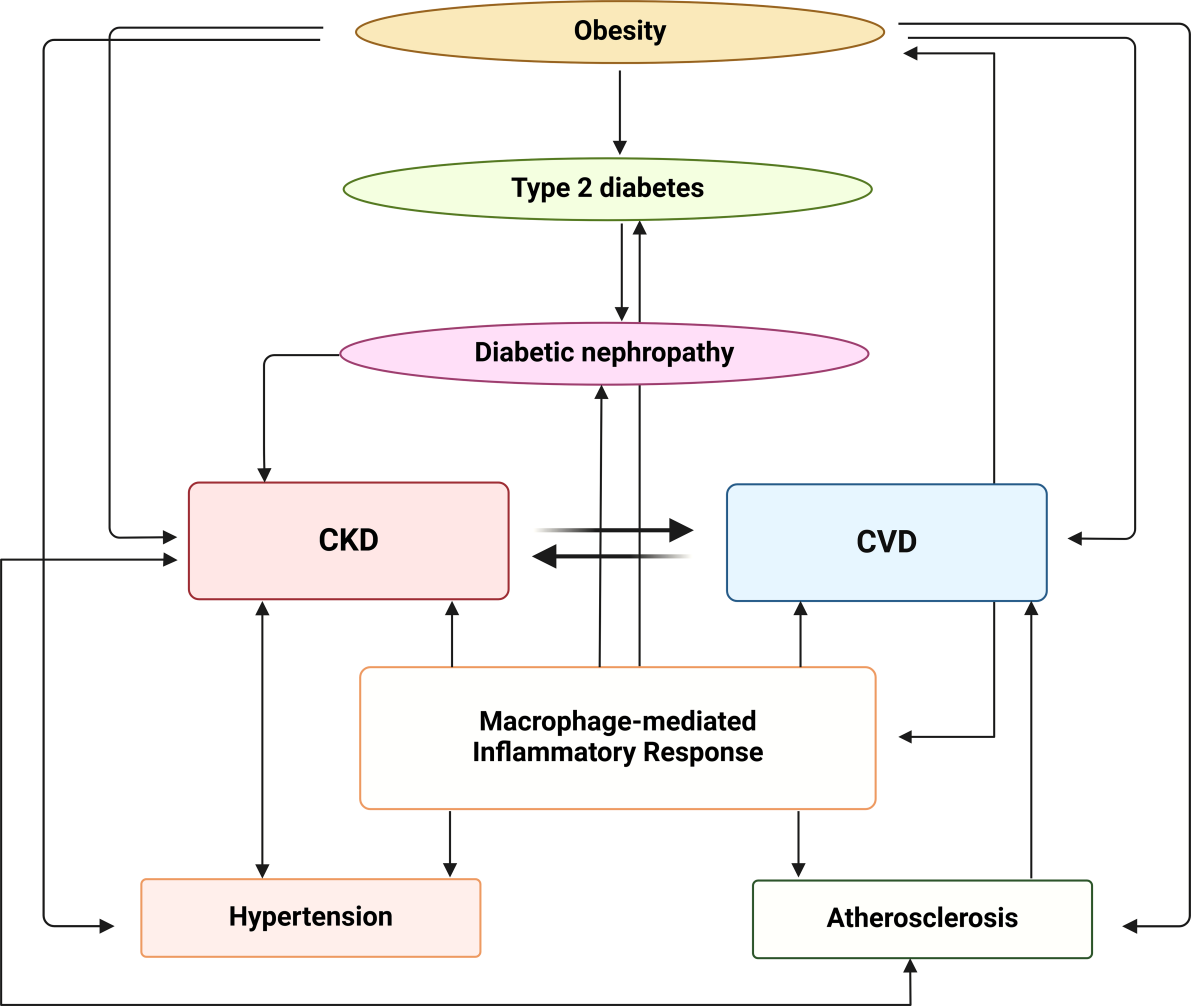
Interplay between metabolic, inflammatory and vascular pathways in the progression of CKD and CVD. Obesity contributes to the development of type 2 diabetes, which can progress to diabetic nephropathy. Diabetic nephropathy, in turn, is associated with both CKD and CVD that have a bidirectional relationship, with each condition exacerbating the other. The macrophage-mediated inflammatory response plays a central role in this network linking CKD, CVD and atherosclerosis, contributing to the progression of both CKD and hypertension. The image was created with Biorender (https://biorender.com).

In summary, macrophages play a key role in the pathogenesis of CKD and CVD associated with obesity (**Figure 4**). Strategies targeting macrophage polarization could be an alternative approach to treat CKD. Numerous agents targeting the JAKs-STAT1/5, JAKs-STAT3/6, TLR4-NF-κB, MAPK, PI3K-AKT-mTOR, AMPK-PPAR and Nrf2-HO-1 pathways have been shown to induce M2 macrophage polarization and/or inhibit M1 macrophage polarization (178, 179), paving the way for the development of novel therapeutic strategies targeting macrophage functions specific to CKD and associated pathologies. However, for the development of novel therapeutic strategies to be successful, certain important parameters should be considered, such as (1) the fact that macrophages may have different functions depending on the stage of M1/M2 plasticity and the dominant macrophage phenotypes in different disease settings, (2) the effects of different therapies, including immunotherapies, on macrophage function, and (3) the transient stage of resident macrophage polarization in the same patients at different stages of kidney disease (180, 181). It therefore becomes clear that in CKD and its associated pathologies, a personalized therapeutic approach is more realistic because it takes into account the type and function of resident and peripheral macrophages of a given patient at a given time point.
